# A Review on Wearable Technologies for Tremor Suppression

**DOI:** 10.3389/fneur.2021.700600

**Published:** 2021-08-09

**Authors:** Julio S. Lora-Millan, Gabriel Delgado-Oleas, Julián Benito-León, Eduardo Rocon

**Affiliations:** ^1^Centro de Automática y Robótica, Consejo Superior de Investigaciones Científicas – Universidad Politécnica de Madrid, Madrid, Spain; ^2^Ingeniería Electrónica, Universidad del Azuay, Cuenca, Ecuador; ^3^Department of Neurology, University Hospital “12 de Octubre”, Madrid, Spain; ^4^Centro de Investigación Biomédica en Red sobre Enfermedades Neurodegenerativas, Madrid, Spain; ^5^Department of Medicine, Complutense University, Madrid, Spain

**Keywords:** neurorehabiliation, pathological tremor, essential tremor, Parkinson's disease, wearable device, assistive technology

## Abstract

Tremor is defined as a rhythmic, involuntary oscillatory movement of a body part. Although everyone exhibits a certain degree of tremor, some pathologies lead to very disabling tremors. These pathological tremors constitute the most prevalent movement disorder, and they imply severe difficulties in performing activities of daily living. Although tremors are currently managed through pharmacotherapy or surgery, these treatments present significant associated drawbacks: drugs often induce side effects and show decreased effectiveness over years of use, while surgery is a hazardous procedure for a very low percentage of eligible patients. In this context, recent research demonstrated the feasibility of managing upper limb tremors through wearable technologies that suppress tremors by modifying limb biomechanics or applying counteracting forces. Furthermore, recent experiments with transcutaneous afferent stimulation showed significant tremor attenuation. In this regard, this article reviews the devices developed following these tremor management paradigms, such as robotic exoskeletons, soft robotic exoskeletons, and transcutaneous neurostimulators. These works are presented, and their effectiveness is discussed. The article also evaluates the different metrics used for the validation of these devices and the lack of a standard validation procedure that allows the comparison among them.

## Introduction

Tremor is defined as a rhythmic, involuntary oscillatory movement of a body part (1). Although physiological tremor is present in everyone, this small degree of tremor is not enough to affect daily activities. However, some pathologies lead to very disabling tremor. Pathological tremor—simply referred to as tremors in the remainder of the document—is one of the most common prevalent movement disorders, affecting over 0.4% of the general population (2), strongly increasing its incidence and prevalence with aging (3). Tremors arise due to various conditions (4), and their exact underlying mechanisms have not been elucidated; thus, none of them is wholly understood (1).

Although there are several causes for tremor disorders, the most prevalent and incident types of tremor arise from two neurodegenerative disorders: Parkinson's disease (PD) and essential tremor (ET) (5–7). ET is the most prevalent pathological tremor (8), affecting 5% of the population over 65 years old (9), while PD has an estimated prevalence of 1% for people over 60 years (10, 11). Other causes for atypical tremors could be multiple sclerosis (12, 13), head trauma (14), and psychogenic tremor (15), among others. Although tremor could not be considered inherently dangerous, more than 65% of the population suffering from upper limb tremor report severe difficulties in performing their activities of daily living (ADL), significantly decreasing their independence and health-related quality of life (16, 17). These patients often present psychological effects due to their condition, such as physical disability (18), leading to social exclusion (19) and depression (20–22). Almost a quarter of patients who go to treatment centers are forced to quit their profession, and 60% decide not to apply for jobs or promotions because of disabling symptoms (23). The exact causes of most of the tremors remain unknown (24, 25), and as they are not curable, the main purpose of the treatments is to alleviate their symptoms (26). Therefore, improving tremor management could drastically reduce direct and indirect costs related to tremor and improve the quality of life and independence of both patients and caregivers.

The treatments for tremor are mainly surgical or pharmacological. In ET, propranolol or primidone is the first-line therapy against tremor, reducing hand tremor by 50% during clinical tests (27, 28). Despite this, up to 30% of patients do not respond to this treatment or experience intolerable second effects (29). Moreover, up to 56% eventually give up their use (30) because of these secondary effects or the lack of efficacy. Regarding PD, levodopa is considered the most effective drug in managing its motor symptoms (31). However, motor fluctuation and dyskinesia seem to be related to levodopa treatment (32); in addition, its effect seems to decrease over the years (33).

Surgical alternatives to pharmacological treatments are stereotactic thalatomy or deep brain stimulation (DBS), which are invasive procedures with an associated high risk. Although both interventions show similar results in managing tremor, DBS is associated with a lower complication rate (34, 35). However, DBS is related to a higher risk of intracranial hemorrhage (4% of patients) (36) and secondary psychiatric effects (37), and, besides, the eligible patient rate is extremely low (1.6–4.5% in PD) (38). High-intensity focused ultrasound (HIFU) has recently emerged as an alternative treatment for medically refractory ET (39). A recent study supports that this noninvasive procedure reduces tremor by 55% after 6 months (40). However, some studies reported tremor recurrence (41) and mild adverse secondary effects such as the alteration in sensation (42) or paresthesia and gait disturbances (41).

Alternative research avenues were explored lately; some studies evaluated the possibility to suppress tremor by modulating afferent feedback to the spinal cord (43), motivated by the noninvasiveness, reversibility, and adaptability of this strategy. However, results were variable within and across subjects (43, 44). This variability is likely due to the complexity of the neural circuits targeted when treatments aim to suppress tremors peripherally in specific muscles (44). Recent works support the idea the stimulation of the afferent pathways through spinal cord stimulation (SCS) may alleviate the symptoms of tremors, probably by disruption of low-frequency synchronization in the corticobasal ganglion circuits (45, 46). Some case reports of SCS treatment for ET (47) and PD (48, 49) have shown its effectiveness in reducing tremor in these patients. These results suggest that this is an exciting area for future research, although its mechanisms remain unknown and it needs to be extensively validated.

In summary, surgery, DBS, and focused ultrasound are effective second-line treatments (50–52). However, they tend to lose effectiveness with time and are invasive procedures that cause nonreversible brain lesions (27). Despite all this variety of treatments, tremor is not effectively handled in 25% of cases (53). In this context, this paper presents the findings of several research works focused on tremor suppression through wearable technologies (exoskeletons and neuroprosthetics devices). These works demonstrated the feasibility of managing upper limb tremors with biomechanical loading, applied through either robotic exoskeletons or transcutaneous neurostimulation. This approach, on the contrary to pharmacotherapy or surgery, suppresses tremors by modifying the limb biomechanics, not targeting their site of origin. This article also evaluates research focused on suppressing tremor by triggering a response either in the central nervous system (CNS) or in the peripheral nervous system (PNS) as a consequence of afferent stimulation.

The paper is organized as follows. First, we describe and classify the different devices that suppress tremor through wearable technologies. Robotic exoskeletons are presented describing classical wearable robotic devices as well as recent soft robotic exoskeletons. Then, we present the use of functional electrical stimulation (FES) to emulate the exoskeleton's effect by using human muscles as actuators of the system. Eventually, we introduce the latest developments for tremor suppression based on afferent neurostimulation. The concept, implementation, and experimental validation are reviewed for all approaches, and then the significant findings are discussed. The high variety of technological approaches that we found highlights the importance of tremor evaluation methods to compare their effectiveness in tremor management, so we dedicate a section to present the most common metrics and discuss their results. The article concludes by outlining current and future research in the field of tremor suppression using neuroprosthetic devices.

## Literature Search Methodology

We conducted a literature search using three different databases: Scopus, Web of Science, and PubMed until March 2021. We used the following in the search query in the title, abstract, and keywords: (tremor) AND (suppress^*^ OR manag^*^ OR reduc^*^) AND {[(robot^*^ OR activ^*^ OR soft) AND (exoskeleton OR orthos^*^ OR neuroprosth^*^)] OR [stimul^*^ AND (electric^*^ OR afferent^*^ OR mechanic^*^ OR vibrat^*^)]}. Besides, we excluded those papers that included the following terms in the title: “surg^*^” OR “deep brain stim^*^” OR “ultrasound” OR “spinal cord stim^*^.” The literature search was limited to papers published in the last 15 years.

Inclusion criteria for this review were as follows:

1. English full-text journal articles or conference proceedings.
2. Studies related to devices for suppressing tremor through different wearable approaches or technologies.
3. Description of the experimental validation and the yielded suppression through quantifiable scales.

Exclusion criteria included the following:

1. Documents that only described the mechanical structure of the device or the design of actuators or new materials intended for tremor suppression.
2. Treatments based on drugs, surgical interventions (like DBS), or noninvasive treatments that do not fulfill the wearability criteria (like transcranial magnetic stimulation or transcranial direct current stimulation).
3. Documents that lacked complete methods, results, or discussion sections.

In those cases that we identified both a conference proceeding version and a full-text journal article of the same study, we only included the complete journal version since it contained further details.

**Figure 1:**
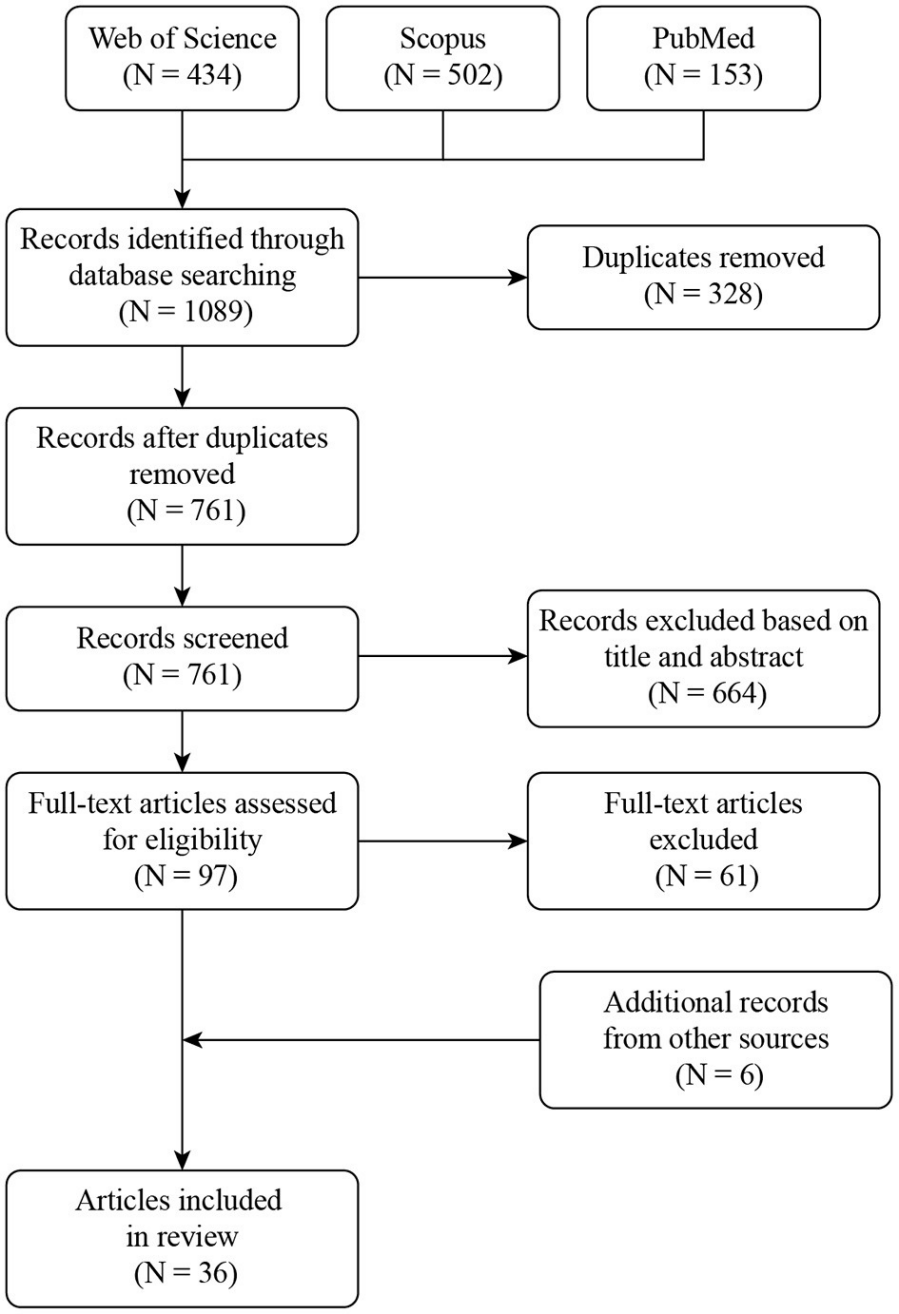
Flowchart of the method used for documents selection.

The initial number of papers (1,089) was reduced to 761 after looking for duplicated documents. After checking the title and abstract, we discarded 664 papers. Finally, 97 were selected for full-text reading. Based on the authors' experience and the bibliography of the reviewed articles, six documents that were not included in the initial search were also selected for full-text reading. As a result, we identified 36 documents out of 1,089 initial records to be considered for this review. Figure 1 shows the flow diagram of the literature search and document selection procedure.

We analyzed several aspects of the selected documents: (i) working principle and hypothesis that supported the tremor management approach for each device; (ii) the experimental setup for device validation; (iii) subject sample size, tremor pathology, and metrics used to quantify the tremor reduction; and (iv) the efficacy of the tremor suppression reported by each approach.

## Results

**Figure 2:**
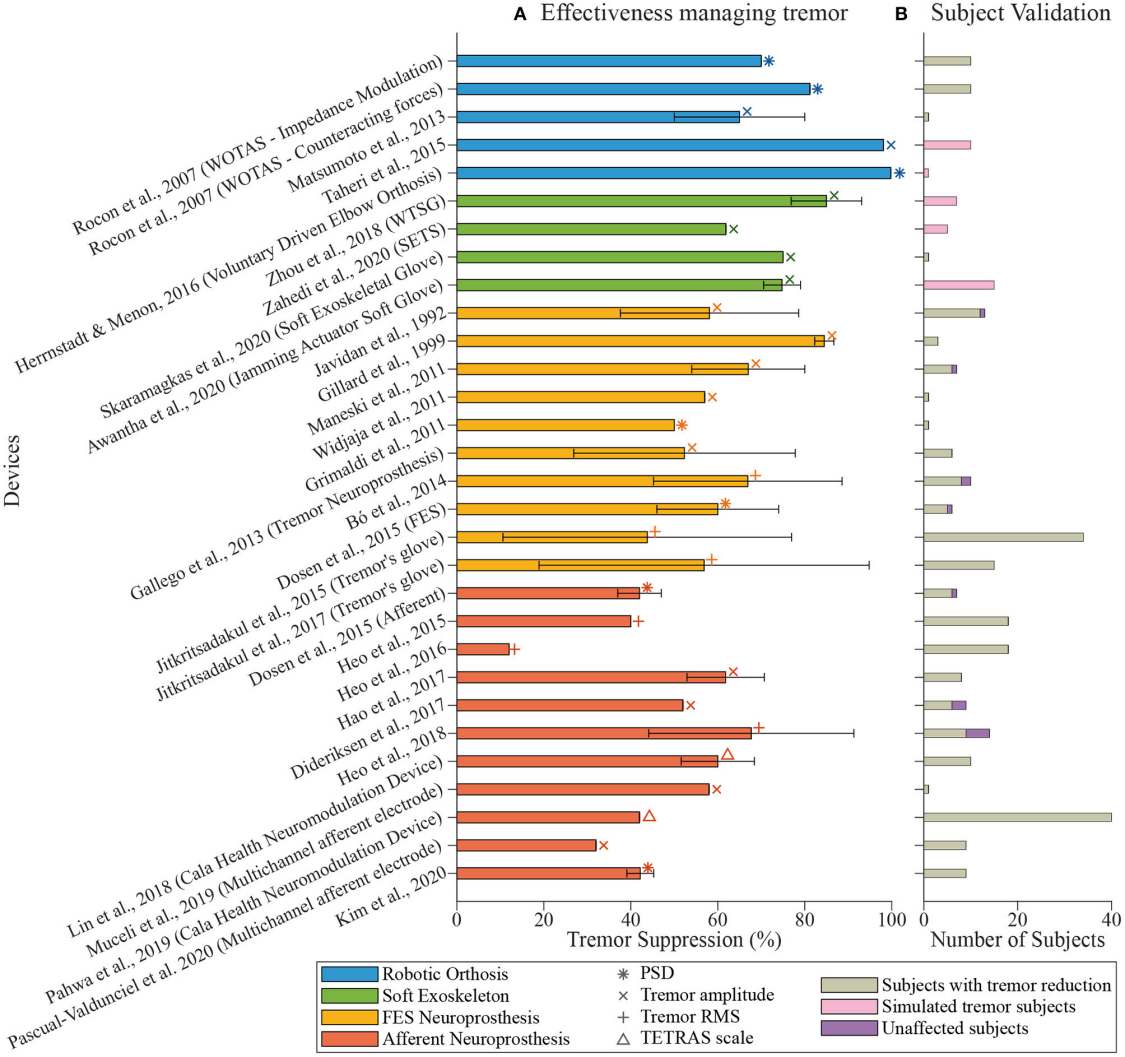
Overview of tremor management devices. **(A)** shows the tremor reduction reported by each paper (percentage, mean ± standard deviation). The color of the bars indicates the technological approach followed by the authors: robotic exoskeletons in blue, soft exoskeletons in green, FES neuroprosthesis in yellow, and afferent neuroprosthesis in orange. Symbols indicate the metric used during the validation of the device. **(B)** shows the number of subjects involved in the validation. Colors point out if the tremor was successfully managed (in gray), if it remained unaffected (in purple), or if tremorous movements were simulated (in pink).

Our literature search led to 36 documents to be considered for this review. Table 1 summarizes the working principle of the reviewed devices, as well as their validation methods and tremor suppression results. In the next subsections, we deepen into the different suppression technologies and their effectiveness as well as the metrics and experimental setups used to evaluate each one of them. Figure 2 represents the devices included in this review, showing their effectiveness in tremor reduction, the metrics, and the number of subjects used during the experimental validation.

**Table 1:** Overview of wearable devices that suppress pathological tremor.

**#**	**Device**	**Suppression strategy**	**Validation method**	**Results**
**Active orthoses**				
*1*	*WOTAS* (54)	Joint impedance control through DC motors	10 tremor-related patients	70% average PSD tremor reduction
		Force application opposed to tremor component using DC motors	10 tremor-related patients	81.2% average PSD tremor reduction
*2*	*Voluntary-driven elbow orthosis* (55)	Motor controller enabled voluntary movements	Replication of 1 ET patient tremor by a mechanical system	99.8% PSD tremor against 1% voluntary movement reduction
*3*	*Electromyogram-controlled exoskeleton* (56)	Motor controller enabled voluntary movements recognized by EMG data	Manual trigger of tremor recognition by 1 ET patient	50–80% tremor amplitude reduction
*4*	*Pneumatic actuation orthosis* (57)	Tremor torque counteracted by pneumatic cylinder	Robotic platform simulating tremor and volitional movements of 10 patients	98.1% tremor suppression at the fundamental frequency (74.3% at second-harmonic)2.08% error tracking voluntary movement
*5*	*Wearable tremor suppression glove* (58)	Forces application through nonstretchable cables	Robotic platform simulating tremor and volitional movements of 7 PD patients	12.4% average error in volitional movement reconstruction
**Soft robotic exoskeletons**				
*6*	*WTSG* (59)	Force application through cable-enabled power transmission	Tremor simulator with seven recorded patient datasets	85 ± 8.1% amplitude reduction, and power reduction for the 1, 2, and 3 harmonics of 87.9 ± 13.6, 92 ± 7.4, and 81.7 ± 13%, respectively
*7*	*SETS* (60)	Force application opposed to tremor movement using magnetic fluid-based flexible semi active actuators	Five healthy subjects simulating tremor	61,82% mean absolute value (MAV) acceleration decreases 58.85% MAV angular velocity decreases 61.89% RMS acceleration decreases 56.22% RMS angular velocity decreases
*8*	*Soft exoskeletal glove* (61)	Force application opposed to tremor movement using PAMs	1 ET patient	75% tremor amplitude reduction and 70% frequency amplitude reduction
*9*	*Soft glove with layer jamming actuator* (62)	Joint rigidity controlled by jamming actuators	Tremor simulator with 15 recorded patient datasets	Maximum amplitude reduction of 74.79 ± 4.23%
**FES neuroprostheses**				
*10*	*Prochazka et al. FES device* (63, 64)	Activation of tremorogenic muscles out-of-phase	3 ET patients; 4 PD patients; six multiple sclerosis patients	58.1 ± 20.5% tremor attenuation (*N* = 12, tremor unaffected in one patient)
*11*	*Gillard et al. FES device* (65)	Activation of tremorogenic muscles out-of-phase	3 PD patients	84.5 ± 2.2% average tremor cancellation
*12*	*Popović et al. Multiple stimulation channels FES platform* (66)	Selective stimulation of multiple muscles out-of-phase	3 ET patients; 4 PD patients	67 ± 13 average tremor amplitude reduction (*N* = 6, tremor unaffected in one patient)
*13*	*Widjaja et al. EMG and FES platform* (67)	Activation of tremorogenic muscles out-of-phase	1 ET patient	57% suppression in tremor amplitude
*14*	*Dosen et al. Tremor predictor based on IHT of EMG and FES platform* (68)	Activation of tremorogenic muscles out-of-phase	2 ET patients; 4 PD patients	60 ± 14% average PSD tremor suppression (*N* = 5, tremor unaffected in one ET)
*15*	*Tremor Neuroprosthesis* (69)	Adaptive cocontraction	2 PD patients; 4 ET patients	52.33 ± 25.48% tremor amplitude reduction (*N* = 26 trials, tremor was exacerbated in 4 trials)
*16*	*Grimaldi et al. FES platform* (70)	Cocontraction	1 ET patient; 1 PD patient; 1 paraneoplastic cerebellar syndrome	50% tremor reduction only in the ET patient
*17*	*Bó et al. FES device* (71)	Isometric cocontraction of the pair of antagonist muscles	10 ET patients	66.9 ± 21.7% tremor RMS reduction (*N*= 8, tremor unaffected and exacerbated in one patient)
*18*	*Tremor's glove* (72, 73)	Constant cocontraction of the pair of antagonist muscles	34 PD patients (72)	43.8 ± 33.2% tremor RMS reduction (61.8% of patients showed at least 30% reduction)
			15 PD patients (and 15 PD patients as sham group) (73)	56.86 ± 37.97% tremor RMS reduction; significantly different from sham group
**Afferent neuroprostheses**				
*19*	*Dosen et al. Tremor predictor based on IHT of EMG and FES platform* (68)	Electrical stimulation under motor threshold	2 ET patients; 4 PD patients	42 ± 5% average PSD tremor suppression (N=5, tremor unaffected in one ET)
*20*	*Multichannel electrode for afferent stimulation* (74, 75)	Out-of-phase sensory electrical stimulation	1 PD patient (74)	58% average reduction in wrist tremor angle
			9 ET patients (75)	32% average reduction in wrist tremor; surface stimulation led to lower reduction
*21*	*Heo et al. electrical afferent platform* (76–79)	Continuous electrical afferent stimulation	18 ET patients, stretched arm task (76)	40% RMS tremor reduction in wrist joint. 60% in MP joint
			18 ET patients, spiral drawing task (77)	12% RMS tremor reduction in MP joint
			14 PD patients (78)	RMS tremor reduction: 67.7 ± 23.6 in finger (*N* = 64%); 62.1 ± 20.0 in hand (*N* = 50%); 53.1 ± 22.9 in forearm (*N* = 71%)
			9 SWEDDs patients (79)	No significant tremor reduction
*22*	*Shanghai Jiao Tong University electrical afferent platform* (80, 81)	Continuous electrical afferent stimulation	2 PD patients (80)	Significant tremor reduction (no data)
			8 PD patients (81)	61.7 ± 8.9% tremor movements reduction 47.9 ± 25.8% EMG reduction
*23*	*Dideriksen et al. electrical afferent platform* (44)	Out-of-phase electrical afferent stimulation	5 PD patients; 4 ET patients	52% average reduction (*N* = 6, tremor unaffected in three patients)
*24*	*Cala Health neuromodulation device* (82–85)	Transcutaneous afferent patterned stimulation of median and radial nerves	23 ET patients (*N* = 10 in treatment group; *N* = 13 in sham group) (82)	60 ± 8.4% tremor reduction in TETRAS scale (spiral drawing)
			77 ET patients (*N* = 40 in treatment group; *N* = 37 in sham group) (83)	Subject-rated Bain and Findley ADL score improvements were greater in the treatment group (49%) than in the sham group (27%). 42% tremor reduction in TETRAS scale
**Afferent neuroprostheses**				
			205 ET patients in three month home therapy (84)	Improvements in TETRAS (62% patients) and BF-ADL (68% patients) scoresWrist tremor reduction in 92% patients (54% patients' improvements greater than 50%)
			15 ET patients (85)	80% of patients showed tremor improvement 60min after the stimulation
*25*	*Kim et al. electrical afferent platform* (86)	Transcutaneous afferent patterned stimulation of radial nerve	9 ET patients	42.17 ± 3.09% PSD reduction
*26*	*Essential platform* (87)	Continuous mechanical afferent stimulation	18 ET patients	Not conclusive
*27*	*Kyushu University Mechanical Vibration Stimulation platform* (88)	TVR movement induction to counteract tremor movement	5 healthy subject	Successful induction of movement through vibrating stimulation

### Wearable Technology for Tremor Suppression

**Figure 3:**
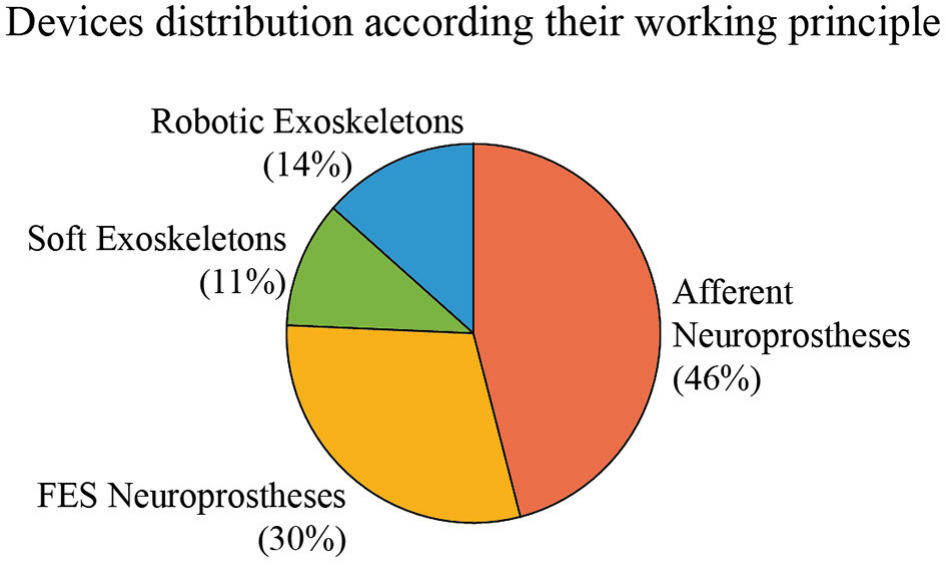
Distribution of the reviewed devices according to their actuation principle. Percentages indicate the proportion of reviewed papers that use each technology.

In this section, we present and describe the devices that claim to manage tremor through wearable technology. We have classified them according to their working principles into robotic exoskeletons, soft robotic exoskeletons, FES neuroprosthesis, and afferent neuroprosthesis. Robotic exoskeletons, or active orthoses, include such robotic devices composed of rigid frames, while soft exoskeletons are composed of flexible elements such as cables or straps. Both kinds of devices base their action on force application and biomechanical loading. Regarding neuroprostheses, FES devices are based on electrical stimulation that produces muscle contraction for biomechanical loading, while afferent neuroprostheses use sensory stimulation to generate a response in the nervous system through the afferent pathways. The intensity of the stimulation in FES devices is always over the motor threshold, while the stimulation in an afferent neuroprosthesis can be low-intensity electrical stimulation (under the motor threshold) or mechanical stimulation. Figure 3 represents the distribution of reviewed papers according to this classification.

#### Robotic Exoskeletons for Tremor Suppression

**Figure 4:**
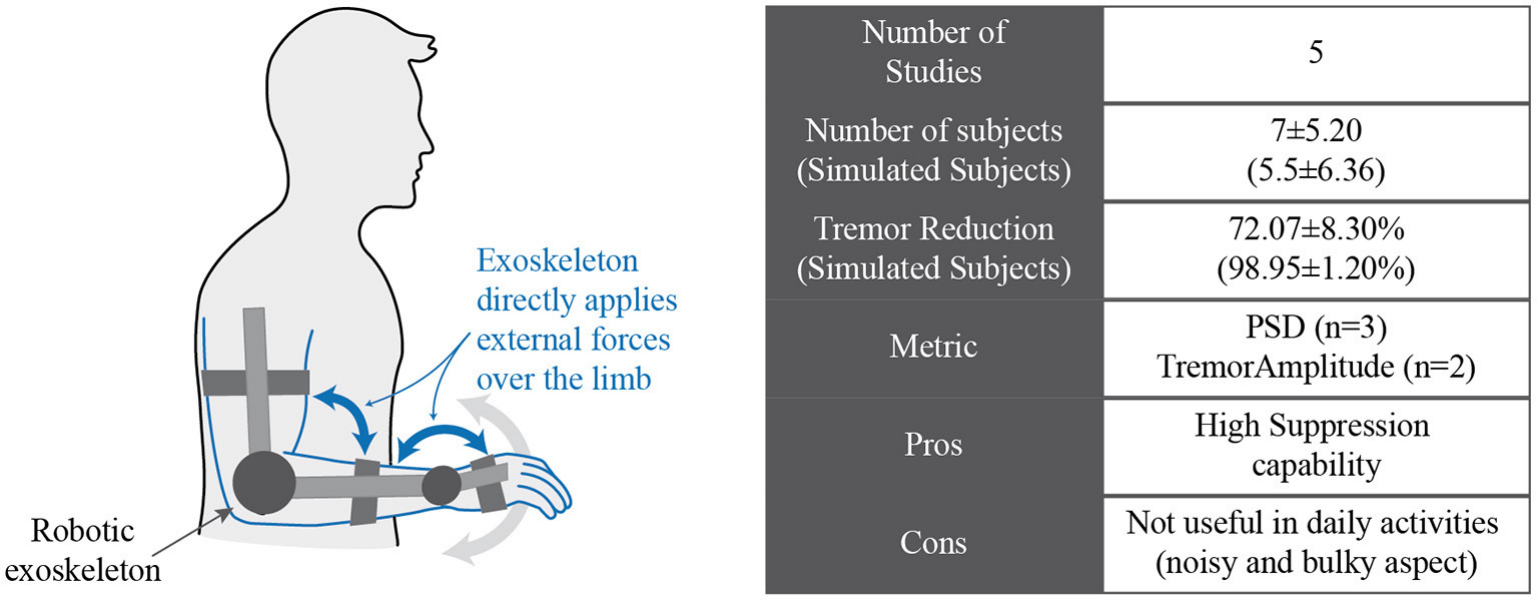
Conceptual representation of a robotic exoskeleton that manages tremor by applying forces over the limb of the subject.

Biomechanical loading is a classical solution for tremor suppression (16). In 1974, Joyce and Rack (89) reported the first results of adding force and inertia to physiological tremor. These results were posteriorly replicated in pathological tremor by adding inertial loads (90–94) or applying forces (95–100) to the affected limb using different kinds of orthoses. Afterward, active orthoses were proposed to achieve these same goals by using actuators; an overview of this technological approach is represented in Figure 4.

One of the first devices that followed this approach was the WOTAS (wearable orthosis for tremor assessment and suppression) exoskeleton reported by Rocon et al. (54). This device was a robotic exoskeleton with three degrees of freedom (elbow flexion–extension, forearm pronation–supination, and wrist flexion–extension) that was able to apply forces to the patient's upper limb joints. This exoskeleton identified the tremor and volitional components of motion with small phase lag by using a two-stage method (101). Once the tremor was identified, WOTAS was able to use two different strategies to counteract tremorous movements: simulating the application of viscosity and inertia to change the impedance of the limb and suppress high-frequency movements (passive control mode) or applying forces opposed to the tremor component of the movement to counteract it (active control mode). Both strategies were tested on 10 tremor-related patients, leading to 70 and 81.2% average power spectral density (PSD) tremor reduction for passive and active control modes, respectively (54).

Alternatively, the paradigm followed by the active elbow orthosis presented by Herrnstadt and Menon (55) was based on reducing tremor by estimating the voluntary movement of the user. The controlled motor of the orthosis only enabled the volitional action, while the tremor movement was rejected. They developed a mechanical system that replicated the movement from an ET patient record to test this device. Using this simulation platform, they reduced the PSD of tremor by 99.8%, while the voluntary movement was reduced to less than 1%.

The same strategy was followed by the exoskeleton developed by the team of Fujie to assist ET patients while eating (56). Their objectives were to identify volitional movement using electromyography (EMG) signals of ET patients in real time and enable only voluntary actions. However, although they were working on it, using an algorithm based on short-time Fourier transformation and time delay neural networks (102), they did not integrate this intention recognition with the robotic exoskeleton. Instead, they tested the tremor suppression simulating this recognition with a switch triggered by an ET patient (56), obtaining that the tremor was reduced by 50–80% compared to not wearing the exoskeleton.

By contrast, instead of generating the volitional movements of the patient, Taheri et al. (57) estimated and canceled the muscle torque responsible for tremor movements. This torque was canceled by generating an equal torque with opposite sense using a pneumatic cylinder. They validated the algorithm with data recorded from 10 patients with severe tremor that was simulated by an artificial wrist joint. Experimental results showed that they were able to suppress tremor movement with an average reduction of 98.1% at the fundamental frequency and 74.3% at the second-harmonic frequency. The average position error on the voluntary movement was 2.08%.

The main limitation of these devices is their poor wearability due to their size and rigid structure (54). Despite attaining a systematic attenuation of moderate and severe tremors, active orthoses were not helpful in daily life as users were reluctant to use them because of their bulky appearance (53).

#### Soft Exoskeletons for Tremor Management

**Figure 5:**
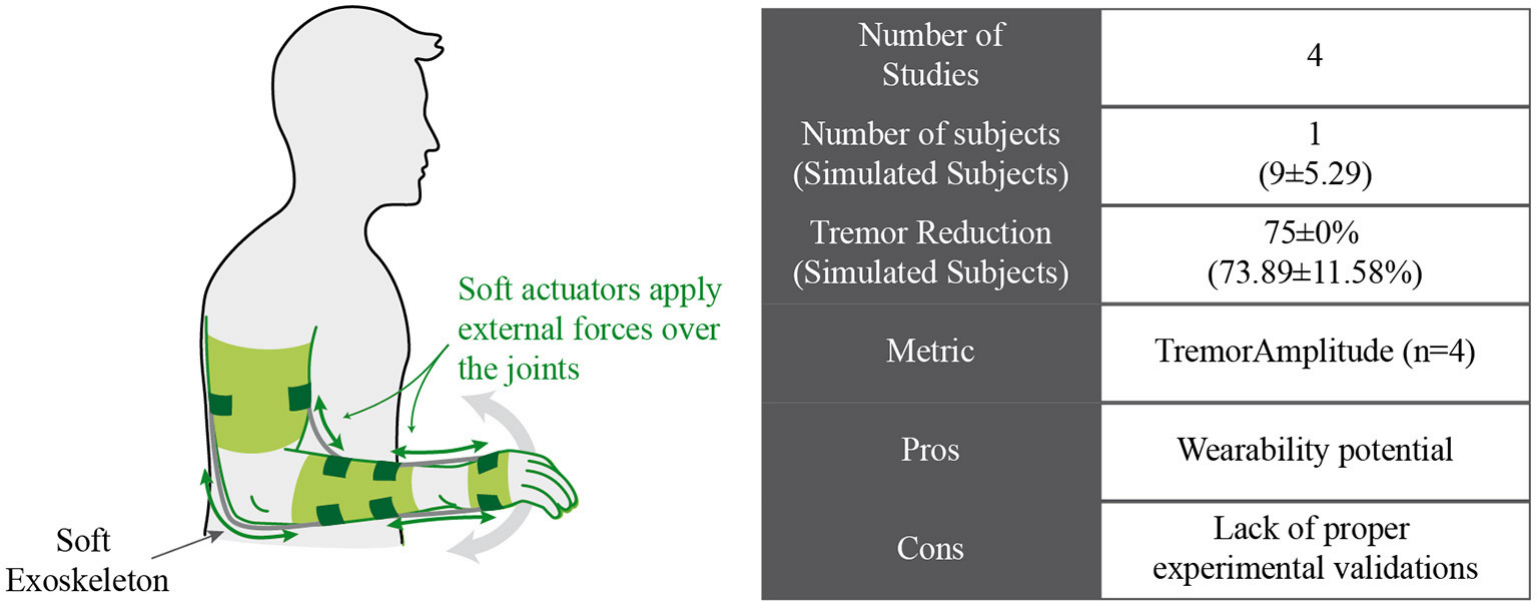
Conceptual approach for soft exoskeletons that manage tremor by applying forces.

New technologies developed in the context of soft robotics enable engineers to create devices more appealing than robotic exoskeletons to reduce pathological tremor while also fulfilling usability requirements for the final users; a conceptual representation of this technology is shown in Figure 5. In this context, Zhou et al. proposed a wearable tremor suppression glove (WTSG) that applied forces to the tremorous hand through nonstretchable cables acting as tendons do (58, 59). These cables were attached to the index finger, thumb, and wrist to suppress tremor in the index finger metacarpophalangeal (MP) joint, thumb MP joint, and wrist joint in the flexion–extension direction. Inertial measurement units were used to sense the system and acquire tremorous and volitional movement, while DC motors coupled with rotary to linear converters were responsible for the actuation of the device.

Primarily, to test this prototype, the authors built a platform to simulate the tremor and volitional movements of seven PD patient. They calculated offline the voluntary action by using a Kalman filter for parkinsonian tremor estimation (103) and used it as the input for their system. They were able to reconstruct the volitional movement of the patients with an average root mean square (RMS) error of 12.4%, with the subsequent tremor suppression. In a second validation, the tremor simulator was also fed with seven PD tremor datasets. The authors evaluated the tremor suppression provided by the WTSG and the device's performance when following voluntary motion (59). The experiments showed an overall tremor amplitude reduction of 85 ± 8.1% and a power reduction for the first, second, and third harmonics of 87.9 ± 13, 92 ± 7.4, and 81.7 ± 13%, respectively. The voluntary motion showed a RMS error for the volitional movement reconstruction of 14.2 ± 2.5% and a correlation coefficient of 0.96 ± 0.01.

Another soft exoskeleton for tremor suppression (SETS) was proposed by Zahedi et al. (60). This device was equipped with a controllable flexible semi active actuator based on magnetic fluid and two hyper elastic blades. The combined action of these two was able to suppress wrist tremor with minimum restrictions on the voluntary motion during flexion/extension, abduction/adduction, and supination/pronation. Five healthy subjects simulated tremor movements in the wrist flexo/extension direction while wearing the device to test this device. These subjects also wore a blindfold, and the authors asked them to keep the movement as constant as possible. After comparing the movement while the system was turned on and off, the results showed that the RMS value of the movement acceleration decreased by 61.89%, and the RMS value of the angular velocity decreased almost 56.22% when SETS was active.

A different approach for a soft device was proposed by Skaramagkas et al. (61), who used pneumatic artificial muscles (PAMs) for suppressing hand tremor of ET patients due to the similar properties of these actuators with those of organic muscles. This device consisted of a PAM linked to target points through tendons, a soft glove that provided attachment points between the PAM and the target points, and a force sensor placed in the contact point to provide feedback of the exerted resistive force.

They tested two prototypes with different application points for the force: the index finger and the metacarpal region. Using the prototypes under open-loop control, the authors obtained the force that provided the maximal decrease in tremor amplitude (89%) and frequency (70%) for one ET patient. By using this force as the set point for a closed-loop controller, both prototypes obtained a maximal reduction of 75% in amplitude and 70% in frequency (61). Although the metacarpal solution provided slightly fewer reductions during closed-loop control, it had the advantage of allowing the free movement of the finger.

Finally, jamming actuators were also proposed as a solution for tremor management. Awantha et al. developed a soft glove for hand tremor suppression based on jamming actuators that stiffened the joint when vacuum was supplied and created resistance to the tremor motion (62). A prosthetic hand simulating finger tremor of 15 tremor patients was used to evaluate this device, while two different combinations of jamming elements and actuator placements were tested. The maximum tremor reduction was obtained for the placement of the actuator in the palmar side, and it yielded to an amplitude reduction of 74.79 ± 4.23%.

The main drawback of this technology is that it is still poorly validated. Except for one of the reviewed works (61), the rest of them only presented experimental validations with healthy subjects or artificial platforms. Thus, there is no objective evidence of the effectiveness of this technology in suppressing tremor.

#### Tremor Suppression Based on Electrical Stimulation Over the Motor Threshold

**Figure 6:**
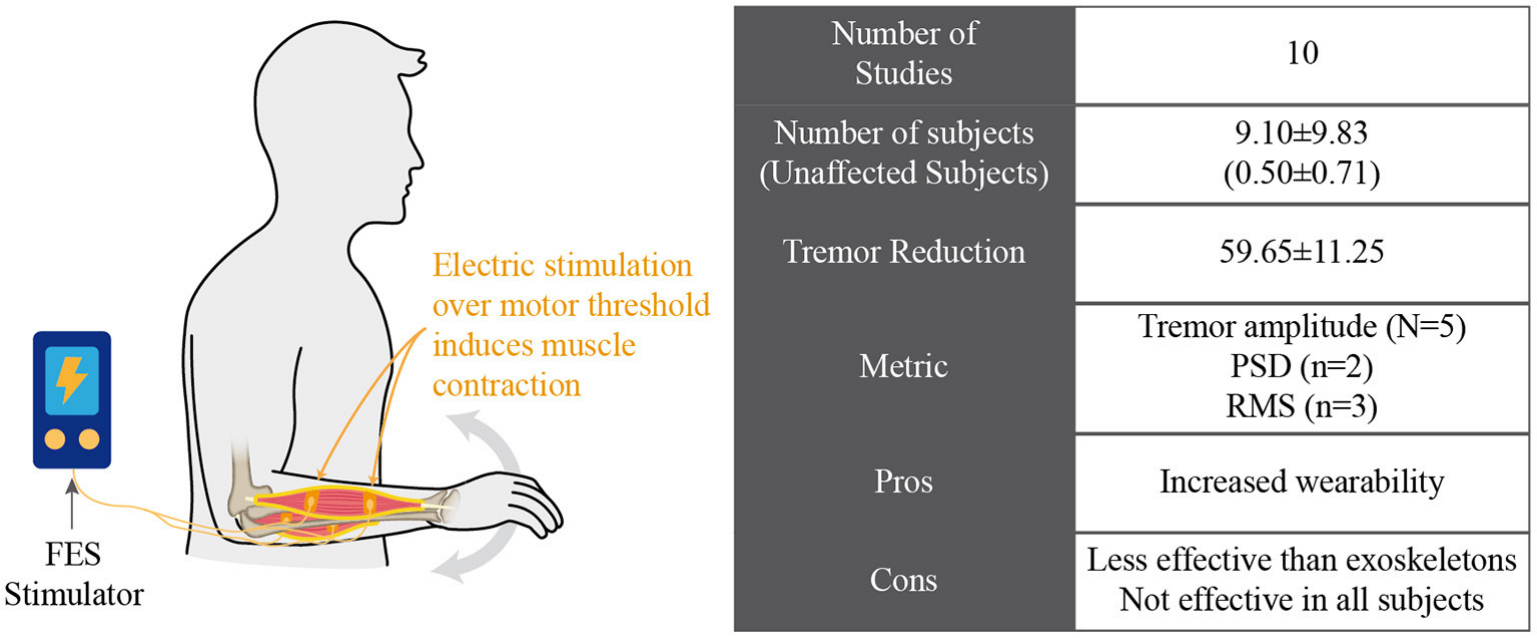
Electrical stimulation for managing tremor by inducing contraction of involved muscles.

Looking for the same biomechanical loading effect provided by active orthosis and soft exoskeletons, some studies have proposed electrical stimulation to reduce and suppress tremor because they enabled smaller and more discreet solutions compared with robotic exoskeletons. FES can activate tremorgenic and/or antagonist muscles to modify the dynamic behavior of the limb or apply forces to counteract the tremorous movements. A conceptual representation is shown in Figure 6. Concretely, the strategies that we detail in this section have the common characteristic that the pulse intensity of the electrical stimulation was high enough to activate muscle fibers and generate a muscle contraction.

Two main strategies have been adopted to yield tremor suppression: cocontraction stimulation and out-of-phase stimulation (44). The cocontraction strategy was based on the stimulation of the pair of antagonist muscles of the affected joint, so the impedance presented by the joint was increased to counteract tremor. On the other hand, out-of-phase strategies applied electrical stimulation to the antagonist of the muscle responsible for the tremorous movement. The amplitude of this stimulation was enough to apply forces that were opposite to those that generated tremor.

One of the first approximations that used FES was the one proposed by Prochazka et al. (63, 64). Their work was based on the out-of-phase activation of the tremorgenic muscles using closed-loop FES to cancel tremorous movements. Their approach was based on that the neuronal activation of tremorgenic muscles generated by the nervous system could be considered as a disturbance that was rejected by the closed loop. However, only the high-frequency tremor-related movements needed to be suppressed, so low-frequency voluntary movements remained unaffected. By properly designing a feedback filter, it was possible to attenuate tremor-related frequencies (2–5 Hz) and minimally affect the frequency range of voluntary movements (0–1 Hz). The device was tested on three patients with ET, four patients with PD, and six patients with cerebellar tremor who presented disabling tremor in the wrist (ET and PD patients) and/or the elbow (in cerebellar tremor). Although tremor in one patient did not decreased, the device achieved an average suppression of 58.1 ± 20.5% (in a range between 91 and 10%) in the rest of the patients.

This same strategy was replicated by Gillard et al. (65), but they used a digital filter instead of analogic circuitry to define the stimulation to be applied to the wrist or finger flexor and extensor muscles. They tested their approach on three PD patients, obtaining an average tremor cancellation of 84.5 ± 2.2%. Afterward, Popović et al. enhanced this approach by developing a FES platform that was able to control multiple stimulation channels for tremor management in multiple joints (66). Their multichannel platform was able to selectively stimulate several single muscles following also an out-of-phase strategy with tunable stimulation properties. This system was tested on seven patients (three ET and four PD); although one of them did not respond to the stimulation, the remaining six showed an average tremor suppression of 67.0 ± 13.0%.

Widjaja et al. proposed a stimulation strategy based on surface EMG and accelerometer information to reduce the delay between tremor detection and stimulation, justifying the inclusion of muscle activation signals because of its earlier generation compared to kinematics information (67). Based on both sensory information, two extended Kalman filters and a phase equalizer algorithm differentiated between volitional and tremor-related components of the movements by calculating the electromechanical delay. They tested this strategy with an ET patient whose tremor was recorded by the accelerometer. The obtained results showed a decrease of 57% in wrist flexion–extension tremor amplitude.

Dosen et al. presented a tremor suppression strategy also based on out-of-phase stimulation of antagonist muscles (68). However, they used the iterative Hilbert transform (IHT) to detect and predict tremor bursts using EMG signals of the muscles involved in tremor generation. The strategy consisted of two consecutive phases: during the first, the system recorded and analyzed EMG signals to detect and predict the timing of tremorgenic bursts. During the second phase, and according to that timing, the stimulation was delivered to the antagonist muscles when the appearance of tremor was predicted on the agonist muscle. They tested this strategy on six patients who presented wrist flexion–extension tremor (four patients due to PD and two patients diagnosed as ET). Although one of the ET patients did not respond positively to this strategy, results showed a tremor suppression rate of 60.0 ± 14.0% for the rest of them when their basal tremor was compared to tremor during out-of-phase stimulation.

A different approach was pursued by Gallego et al. (69), who designed a neuroprosthesis to generate mechanical loads in a pair of antagonist muscles in such a way that the impedance of the joint was properly manipulated artificially, cocontracting the muscles involved in the tremorous movement. As the dynamic response of the muscles to the tremor movement is analog to a low pass filter, by artificially increasing the stiffness and viscosity of the joint, the cutoff frequency would be decreased. Consequently, if this frequency is over the tremor frequency, tremorous movements would be filtered out. The system identified the tremorous and voluntary components of the movement and adapted the level of elicited cocontraction to the instantaneous frequency and amplitude of the tremor. This neuroprosthesis was validated within two PD patients and four ET patients, who reported a reduction of 52.3 ± 25.5% in 26 out of 30 trials compared to trials where the neuroprosthesis was not active.

Grimaldi et al. (70) also evaluated this same strategy in one PD patient, one ET patient, and one paraneoplastic cerebellar syndrome. However, they only reported a successful tremor reduction in the ET patient, whose tremor decreased 50% during the stimulation. Bó et al. also developed a neuroprosthesis based on the cocontraction strategy (71). They stimulated in an open-loop configuration, turning it on and off while subjects performed a static motor task, and validated this strategy in 10 ET patients. Although one of these patients did not clearly enhance his tremor amplitude and other even increased it, the other eight patients returned positive results, reducing the tremor amplitude by 66.9 ± 21.7%. However, these patients showed different behaviors when stimulation was applied, presenting in some cases an adaptation phase before the tremor was effectively managed.

Jitkritsadakul et al. (72, 73) also delivered constant electrical stimulation over the motor threshold on hand muscles to suppress tremor, and they also considered the hypothesis of interfering with the cerebello-thalamo-cortical circuit through afferent stimulation. During their first approach (72), they compared the tremor angular velocity before and during stimulation in 34 PD patients, and their results showed an average improvement of 49.6 ± 38.89% in the peak amplitude and an average reduction of 43.8 ± 33.2% in the RMS value of tremor. However, just 70.6 and 61.8% of patients showed at least 30% tremor suppression in the peak amplitude and RMS value, respectively. Later, they developed and validated the Tremor's glove device to detect and suppress tremor based on this same strategy (73). They compared the tremor evolution in 30 PD patients wearing the Tremor's glove device (*N* = 15) or a sham replica (*N* = 15). Their results pointed out that the device significantly managed tremor in the glove group compared to the sham group according to the reduction in the RMS and peak value of the angular velocity (56.86 ± 37.97% X-axis and 49.64 ± 71.48% Y-axis, respectively) and the Unified Parkinson's Disease Rating Scales (UPDRS; 1.47 ± 0.74).

Despite these promising results, there are several drawbacks inherent to this technology. Timing of the control and selectivity of muscle stimulation are crucial aspects for tremor management (76). Besides, muscle fatigue due to induced contraction also decreases the effectiveness of these devices (66).

#### Stimulation of Afferent Pathways for Tremor Management

**Figure 7:**
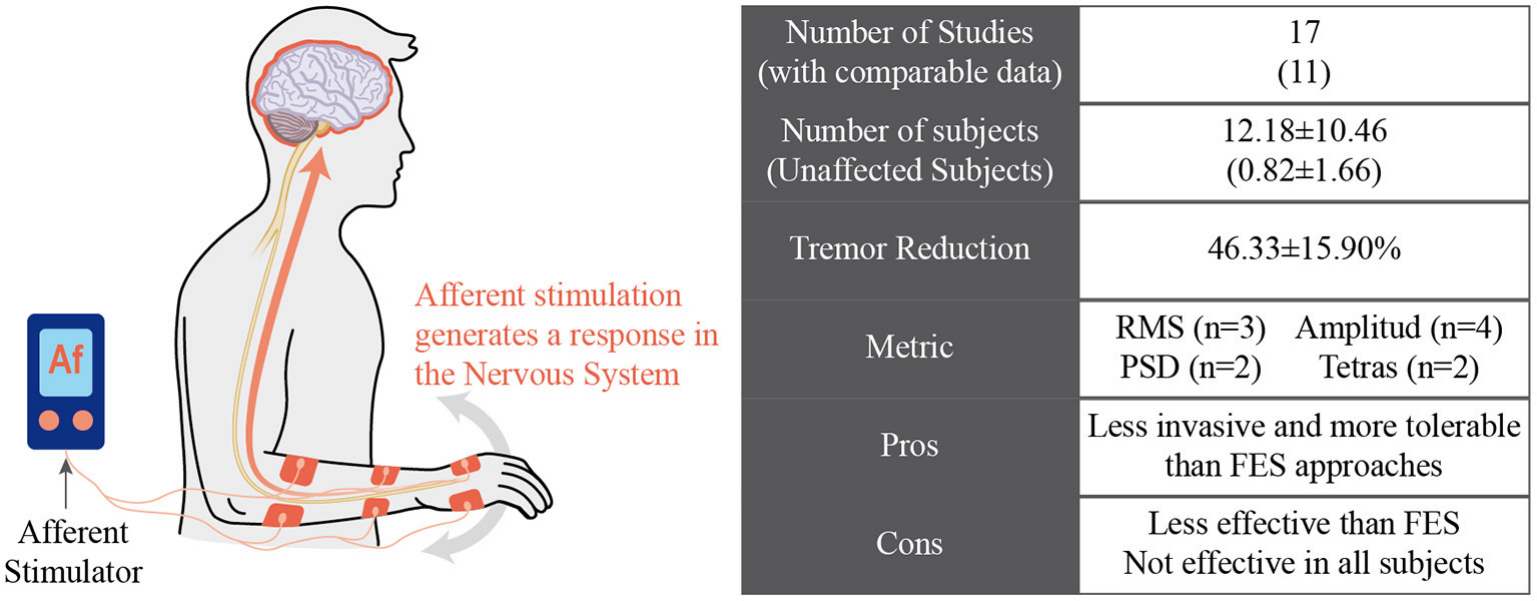
Afferent neuroprosthesis manages tremor by inducing a response in the nervous system of the subject.

Several studies have found relationships between tremor generation and sensory activity to circumvent the limitations of FES-based tremor suppression. For example, providing proprioceptive input through passive joint movements can modulate tremor in PD patients (104). Similarly, low-level electrical stimulation applied at the wrist joint modulated the tremor frequency (105). In this way, sensory or afferent stimulation generates a response in the CNS that can modify tremor in patients. Figure 7 schematically illustrates this approach.

Based on these studies, Dosen et al. presented the hypothesis that tremor could be reduced by stimulating sensory pathways instead of activating muscle fibers (68). Their work compared the effect of stimulation above and under the motor threshold on the tremor of four PD and two ET patients. Results for motor stimulation are presented in the previous section of this document, achieving an average tremor reduction of 60 ± 14%. They performed the same protocol with the same patients to test the sensory stimulation but using lower stimulation levels that did not generate muscular activity. Sensory stimulation resulted in an average tremor suppression of 42.0 ± 5.0%; although lower than that yielded with motor stimulation, it was postulated as a feasible alternative for tremor management (68).

In (44), Dideriksen et al. tested both surface and intramuscular stimulation and analyzed the most convenient stimulation settings (pulse amplitude and timing) to reduce the tremor amplitude. They recruited five PD and four ET patients who were stimulated according to the algorithm proposed by Dosen et al. (68) and received the stimulation through one of the tested electrode interfaces. For each patient, the different stimulation parameters (intensity and burst duration) were varied systematically. Although most patients (66.67%) showed a significant tremor reduction, with an average magnitude of 52% in the best case for each patient, the optimum conditions for tremor reduction varied between patients, pointing out the potential utility of patient-specific stimulation protocols.

As a second step, these same authors developed a multichannel electrode for muscle recording and stimulation (74). They tested this electrode using the same protocol as in (68) in one PD patient following the out-of-phase electrical sensory stimulation strategy. The patient showed an average tremor angle reduction of 58%, in the same attenuation range reported in their previous study. This technique was also assessed during a broader study (75) that involved nine ET patients. Results from this study pointed out that the use of this intramuscular electrode for out-of-phase electrical afferent stimulation led to a 32% average acute tremor reduction, significantly higher than the reduction achieved by surface electrodes.

A similar approach was followed by Heo et al., who studied the effects of electrical afferent stimulation in ET patients (76, 77). Sensory electrical stimulation was delivered to 18 ET patients on four upper limb muscles (flexor carpi radialis, extensor carpi radialis, biceps brachii, and triceps brachii) while the velocity of MP and wrist joints were measured. Two experimental setups were considered at three different phases (prestimulation, during stimulation, and 5 min poststimulation): (1) arms stretched forward during 15 s (76) and (2) Archimedes spiral drawing (77). By comparing the angular velocity before and during stimulation, electrical sensory stimulation resulted in a reduction ratio of RMS angular velocity for MP (60%) and wrist joints (40%) during the arm stretching task (76) and for MP joint (12%) during the spiral drawing task (77). These reductions were also measurable 5 min after the stimulation was applied in both experimental setups.

These same authors also tested their approach in 14 PD patients (78) and nine patients with scans without evidence of dopaminergic deficit (SWEDDs) (79). The tremor of these patients was evaluated during resting tasks before, during, and 5 min after the sensory stimulation by using the RMS of the angular displacement of the index finger, hand, and forearm. Although their strategy did not significantly reduce the tremor in SWEDDs patients (79), a variable percentage of PD patients (between 50 and 71% depending on the segment) reported a reduction in tremor amplitude ranging from 53 to 68% during stimulation (78). Five minutes after the stimulation, this suppression effect was still measurable in some patients (between 57 and 71% depending on the segment) with a reduction ratio ranging from 56 to 60%.

Based on a similar principle, Hao et al. hypothesized that electrical afferent stimulation could affect the transmission of tremorgenic signals, inhibiting tremor in PD patients as a consequence (80, 81). To test this hypothesis, they applied surface electrical stimulation on the dorsal skin of the hand, near the MP joint of the index finger. A preliminary study significantly reduced wrist and elbow flexion tremor and forearm pronation tremor in two PD patients (80). Lately, in a broader study, eight PD patients with tremor dominant symptoms were stimulated using an amplitude fixed from 1.5 to 1.75 times the radiating threshold (the stimulus amplitude that produces a radiating sensation from the dorsal skin to the fingers). Although tremulous movements and EMG signals seemed to be increased in some trials due to the stimulation, both metrics decreased their severity in most cases, resulting in an average peak spectral amplitude reduction of 61.6 ± 8.9% and an average EMG activity reduction of 47.9 ± 25.8%.

A different approach was followed by the team led by Pahwa (82–84): they applied bursts of noninvasive electrical stimulation alternately to the median and radial nerves of the wrist at a frequency tuned to the tremor frequency of the wearer. They hypothesized that this stimulation would modulate the ventral intermediate nucleus and, therefore, would reduce the tremor in ET patients. These authors conducted three different studies to assess the effect of this strategy after 40 min of stimulation in ET patients. Twenty-three patients participated in a study that showed a 60 ± 80.4% tremor reduction in the spiral drawing Tremor Research Group Essential Tremor Rating Assessment Scale (TETRAS) score for the treatment group (*N* = 10) compared to the sham group (*N* = 13) (82).

In the same way, in (83), the treatment group (*N* = 40) reported more significant improvements in the subject-rated Bain and Findley Activities of the Daily Life (BF-ADL) score (49%) than the sham group (27%, *N* = 37). The authors also evaluated the effects of this stimulation during a 3-month therapy in (84). A total of 205 ET patients were instructed to use the therapeutic device twice daily at home, and most of them (92%) improved their tremor according to accelerometer measurements at the wrist, with 54% experiencing an improvement greater than or equal to 50%. In addition, the clinician-rated TETRAS score was improved in 62% of patients, and the patient-rated BF-ADL was also improved in 68% of patients. Finally, the authors analyzed the duration of the effect of this stimulation in (85). They followed the same stimulation treatment as in previous studies with 15 ET patients and found that for 80% of them, the suppressive effect of the treatment lasted for 60 min at least.

Radial nerve stimulation to manage tremor in ET patients was also used by Kim et al., who developed a wearable device that assessed tremor in real time and tuned the stimulation parameters according to open-loop or closed-loop paradigms (86). This device was tested with nine ET patients who showed an overall tremor power reduction of 42.17 ± 3.09%. However, not all trials showed significant tremor reduction. Besides, they noticed that different stimulation parameters affected the attained reduction, so they should be properly tuned to manage tremor successfully.

Based on the results obtained by these electrical afferent stimulation devices, Lora-Millan et al. (87) evaluated a new hypothesis to suppress tremorous movements in ET patients by using mechanical afferent stimulation instead of low-level electrical stimulation. Their work was based on the hypothesis that sensory responses from Pacinian corpuscles could provide a pathway to modulate the circuits that mediate tremor in ET. These authors used piezoelectric actuators to stimulate the fingertips, palm of the hand, and anterior forearm with mechanical vibration at different frequencies. They tested this hypothesis over 18 ET patients who performed the same postural task to trigger the tremor, keeping their most affected arm on a support, with the forearm, hand, and fingers outstretched against gravity. Although the dominant trend in tremor response was to increase, the high variability observed in tremor severity, even without stimulation, made it difficult to interpret the results and, therefore, to reach conclusions.

Another mechanical stimulation approach was also proposed by Liu et al. (88), although they aimed to induce movements to the tremorous limb to counteract the tremor. They applied mechanical vibration over the pronator teres and supinator muscles to induce sustained muscle contraction, referred to as tonic vibration reflex (TVR). In this work, they proposed to use TVR to induce a movement that would counteract the tremorous movement in ET patients. To validate this approach, they induced a periodic pronation–supination movement in five healthy patients by using mechanical vibration. However, they did not present a proper validation counteracting tremorous movement in real patients.

Although these works present promising results, not all patients respond successfully to the afferent strategy for suppressing tremor, and the mechanisms that mediate their effects are not fully understood. In addition, as the physiopathological hypothesis that supports each device is different (106), it is difficult to compare their effectiveness.

### Metrics in Tremor Assessment

Assessing tremor and its possible reduction is crucial to evaluate the effectiveness of the systems for tremor management. In clinical practice, motor symptoms and motor complications are most commonly appraised during clinic visits by rating the performance on clinical scales (e.g., UPDRS, TETRAS, Fahn-Tolosa-Marin). This clinical assessment is subject to bias from placebo effects, anxiety, or the opposite “white coat syndrome,” where patients apply an extra effort, resulting in a performance that does not fully reflect patients' abilities (107, 108). To address this issue, handwriting and drawing patterns are often used to quantify tremor from a clinical perspective (109). Recording such patterns using a digitizing tablet has been introduced as one way to provide precise quantification (110). An example of this approach is the metric developed by the Tremor Research Group to quantify the severity of ETs and their impact on ADL and TETRAS (111). This scale was developed to merge clinical and technical quantification of tremor. It has excellent face validity, interrater reliability, and sensitivity to change. It was adopted in the studies proposed by Cala Health to evaluate the performance of their tremor suppression neuromodulation device (82–85).

From the point of view of tremor quantification, the two most important factors are frequency and amplitude. In this regard, the advances of wearable sensing technologies, in particular inertial measurement units (112), enable the development of different metrics to quantify tremor and assess the electiveness of the technologies proposed. These are the different metrics proposed to evaluate the tremor suppression achieved by the systems considered:

**Tremor amplitude:** This metric is the most used by the works reported in this review; it compares the maximum tremor amplitude before, during, and after the treatment. Of the 30 reviewed articles, 14 adopted the reduction of the tremor amplitude as a metric.
**RMS:** The RMS value is the most relevant measure of the amplitude of a tremor signal because it considers its history and provides a value directly related to its energy content. Therefore, this metric's evolution is directly related to the ability of the device to reduce tremor. Moreover, this measure allows taking data, both positive and negative, and obtaining a more exact metric. Of 30 articles, six adopted this metric.
**PSD:** Tremor is well suited to spectral analysis, the most popular method of tremor quantification, because of its oscillatory characteristics (113). It is used to calculate the PSD function indicating the signal power at different frequencies across the spectrum. The dominant frequency of tremor is evident as a peak in the PSD, while the average tremor amplitude can be determined from the area under the peak (114). In the tremor analysis, it refers to the magnitude of the most recurrent frequencies at the time of measurement, allowing observation of a decrease in tremor. Of the 30 studies, seven used this metric to evaluate.

A significant limitation we encountered in most of the studies was that the effectiveness of tremor suppression based on the metrics mentioned above was mainly based on trials with a short duration of time. For example, the experimental trial duration in (76) was only 15 s, or if longer trials were used as in (44, 68), the authors divided them into epochs. This methodology tried to cope with the high intrinsic variability of tremor (44, 54, 68, 76), but this may not be effective if several minute trials were considered (87). This is a relevant issue to face during the experimental validation of these technologies because of the high fluctuations in tremorous movements (44, 87), which even could be caused by subjective factors such as anxiety, distraction, or surprise (44, 112, 115, 116).

## Discussion and Conclusions

This paper reviewed and discussed the concept of tremor suppression using wearable technology (summarized in Table 1). We identified four groups of technological approaches for tremor management: (1) active orthosis or robotic exoskeletons, (2) soft robotic exoskeletons, (3) FES neuroprosthesis, and (4) afferent neuroprosthesis. Although all reviewed works claimed to manage tremor effectively, there are different degrees of effectiveness, as illustrated in Figure 2.

The technology that achieved the most significant reduction corresponds to active orthosis (exoskeletons). However, several limitations in wearability and comfort have yet to be addressed. Despite their effectiveness, users considered that they hampered their social relationships because of their bulky aspect, noise, and size. In addition, load transmission from the exoskeleton to the human musculoskeletal system was highly inefficient and was an issue to face (53). In summary, robotics-based solutions have shown clinical evidence of the approach based on human limb impedance control. However, it resulted in bulky and noncosmetic solutions for which patients were especially reluctant.

New approaches based on soft actuators are postulated as the next step in the development of this kind of device. These soft technologies could potentially increase the wearability of the resulting device and therefore increase its usability and reduce user rejection. However, further research is required to develop new soft actuator technologies in terms of cosmetic and aesthetic (low weight, compact to be worn beneath the clothes) and functional requirements (torque and bandwidth). As a result, there is yet a lack of proper validation of these actuators as a feasible solution for tremor management. In fact, only the soft exoskeletal glove presented by Skaramagkas et al. (61) presented a validation involving one actual ET patient.

Despite the large variety of robotic devices, their efficacy largely relies on their actuation mechanisms; however, this is not the only factor that interferes with the performance of a robotic exoskeleton. Sensory systems, control strategies, and human factors are also determinants of the efficacy of robotic exoskeletons. Human factors such as adaptation of the user to the orthoses structure, concrete characteristics of the tremor, or individual biomechanical properties condition the performance of these devices.

Some researchers, also focusing on increasing the wearability of these devices based on biomechanical loading, evaluated the use of electrical stimulation over the motor threshold to induce muscular contractions and generate forces or modify the biomechanics of the tremorous limbs. These devices have proven to be effective in suppressing tremor, although their effectiveness was lower than for robotic exoskeletons. Despite the promising results, several drawbacks are challenging to address. Regarding its control, electrical stimulation over the motor threshold requires precise real-time synchronization for reliable performance. The synchronization of the muscle activation timing with the tremor is crucial for proper tremor management. Possible time delays due to the control loop could reduce or avoid the effect of FES stimulation (76). Besides, the dependency between the control algorithm and the properties of the musculoskeletal system could lead to instability or undetermined states because of changes in muscle conditions. Selectivity of muscle stimulation is also an aspect that requires additional research for a proper operation of the systems.

Recent works support the idea that stimulation of the afferent pathways may alleviate the symptoms of tremors. Several groups developed neuroprosthesis focused on this concept, aiming to be less invasive and more tolerable by users than FES devices. However, their effectiveness was lower, and some patients did not respond to this treatment. Another drawback that hampered a direct comparison between tremor management results is the fact that each of the different devices described in the literature was based on a different physiopathological hypothesis (106). More profound studies are required to properly characterize the interaction between the afferent pathways and the neural structures involved in tremor generation. A complete understanding of these interactions would lead to a more efficient tuning of the stimulation strategies to the concrete characteristics of each pathology.

Another critical limitation that hampers a proper evaluation of these alternative treatments is the high variability in the metrics to quantify tremor reduction. There is an evident lack of a standard procedure to evaluate tremor management with wearable technologies, making it difficult to compare results from different works. Differences in several aspects like the postural task, duration of the trial, or variation in the experimental conditions hamper extracting conclusions when comparing devices that followed different experimental procedures. This is particularly important because of the high intrinsic fluctuations of tremor movements (44, 87). For instance, several works used static tasks to trigger tremor and assess tremor management. However, this procedure could not represent daily living activities (117), and therefore, the experimental validation could not be useful to evaluate the effect on assisting the patient in daily life. In this sense, only the Cala Health neuromodulation device reported its effect over the performance of activities of the daily life (82–85).

Clinical validations of these technologies are still in the early stages, as the clinical evidence for their effectiveness is mainly based on a limited number of patients. Except for the Cala Health neuromodulation device, which reported experimental validation with 40 (83) and 205 (84) ET patients, and the Tremor's glove, which reported a validation with 34 PD patients, the rest of the reported devices are validated with less than 20 patients. Therefore, there is still a lack of large clinical trials to consider these technologies as a clinical alternative for tremor management.

Further, also the Cala Health's device and the Tremor's glove are the only two devices that reported experimental validations with a control group that used a sham version of the device (73, 82, 83). Since the validation process also needs to face the problem of high tremor variability (44, 87, 112, 115), further research that compares the action of these devices with sham controls would ensure the effectiveness of these devices.

In summary, this paper reviews the different approaches based on wearable technologies to suppress pathological tremors. We analyzed the complete spectrum of recent developments, from bulky active orthoses, which provide high suppression rates but are not feasible in real life, to new approaches such as (1) soft robotic exoskeletons, (2) FES, or (3) afferent neuroprosthesis. These current developments aim to attain more discrete and wearable solutions, although their effectiveness is usually lower when compared to exoskeletons.

Promising results derived from these devices illustrate their ability to suppress tremor, although they lack the functionality to represent an alternative treatment for tremor. There is no research focused on using these devices in combination with pharmacological or surgical tremor treatments. Researchers should evaluate the ability of these technologies to complement traditional tremor treatments. They have the potential to reduce medication intake or to prolong the effectiveness of surgical tremor treatments.

Further research is required to transform these devices in a real stand-alone alternative treatment for tremor. (1) Although soft actuators seem to be an alternative for wearable solutions, their tremor-suppressing potential needs to be validated with real patients. (2) FES or afferent neuroprosthesis should be extensively validated on larger samples of patients, including control and sham populations, before being considered a clinical alternative for tremor suppression. (3) A standard benchmark for testing and validating these devices, including metrics that account for tremor fluctuations, should be defined and developed. These developments would help researchers to compare different alternatives and find the best technological approach for tremor suppression for each patient.

## Author Contributions

JL-M designed and performed the main literature review, collected the information about the different devices, and drafted and wrote the manuscript. GD-O collected the information about the different devices and drafted and wrote the manuscript. JB-L revised the manuscript. ER designed the review, revised the draft, and made substantial comments. All authors approved the final manuscript.

## Conflict of Interest

The authors declare that the research was conducted in the absence of any commercial or financial relationships that could be construed as a potential conflict of interest.

## Publisher's Note

All claims expressed in this article are solely those of the authors and do not necessarily represent those of their affiliated organizations, or those of the publisher, the editors and the reviewers. Any product that may be evaluated in this article, or claim that may be made by its manufacturer, is not guaranteed or endorsed by the publisher.
